# Perception, acceptability and challenges of digital adherence technology among TB healthcare workers

**DOI:** 10.5588/pha.24.0008

**Published:** 2024-06-01

**Authors:** I. Gordon, B. Odume, C. Ogbudebe, O. Chukwuogo, N. Nwokoye, S. Useni, E. Efo, M. Gidado, E. Aniwada, A. Ihesie, D. Nongo, R. Eneogu, O. Chijioke-Akaniro, C. Anyaike

**Affiliations:** ^1^ KNCV Tuberculosis Foundation Nigeria, Abuja, Nigeria;; 2 KNCV TB Plus, The Hague, Netherlands,; 3 Department of Community Medicine, University of Nigeria Teaching Hospital, Enugu,; 4 United States Agency for International Development (USAID), Abuja,; 5 National Tuberculosis, Leprosy and Buruli Ulcer Control Programme, Abuja, Nigeria

**Keywords:** HCWs, DAT, Nigeria, tuberculosis

## Abstract

**INTRODUCTION:**

Successful treatment of TB requires high levels of adherence to treatment. This has been found to be below optimal with directly observed therapy (DOT), and digital adherence technologies (DATs) offer a promising approach to non-adherence to medication and improving treatment outcomes. This study explores the perception, acceptability, and challenges of DATs among healthcare workers (HCWs).

**METHODS:**

The study was conducted in eight states in Nigeria among Health Care workers involved in treating patients with TB. This was a descriptive cross-sectional study using an open questionnaire and analysed using IBM SPSS v25.

**RESULTS:**

Twenty-three HCWs (95.8%) agreed that DATs helped them provide better support and counselling to their patients. All of them would recommend DATs to their patients and found it easy to explain them. Eleven (45.8%) of them were not able to use DATs on a few occasions; their reasons were poor network (*n* = 9, 37.5%) and (*n* = 1, 4.2%) power failure.

**CONCLUSION:**

DATs help HCWs provide better support and care regarding real-time tracking of their patients’ adherence to treatment and possibly reduction of attrition. This implies that DATs are a suitable alternative to DOT to help HCWs provide the best care and support to their patients towards achieving the End TB targets.

## Abstract (fr)

**INTRODUCTION:**

Le traitement réussi de la TB nécessite des niveaux élevés d'observance du traitement. Cela s'est avéré inférieur à l'optimal avec le traitement sous observation directe, et les technologies d'observance numérique (DAT) offrent une approche prometteuse de la non-observance des médicaments et de l'amélioration des résultats du traitement. Cette étude explore la perception, l'acceptabilité et les défis des DAT chez les travailleurs de la santé (HCW, pour l’anglais « *healthcare worker* »).

**MÉTHODES:**

L'étude a été menée dans huit États du Nigeria auprès de travailleurs de la santé impliqués dans le traitement des patients atteints de TB. Il s'agissait d'une étude transversale descriptive utilisant un questionnaire ouvert et analysée à l'aide d'IBM SPSS v25.

**RÉSULTATS:**

Vingt-trois HCW (95,8%) ont convenu que les DAT les aidaient à fournir un meilleur soutien et des conseils à leurs patients. Tous recommandaient les fichiers DAT à leurs patients et trouvaient facile de les expliquer. Onze d'entre eux (45,8%) n'ont pas pu utiliser les fichiers DAT à quelques reprises, en raison d'une mauvaise qualité du réseau (*n* = 9 ; 37,5%) et d'une panne de courant (*n* = 1 ; 4,2%).

**CONCLUSION:**

Les DAT aident les HCW à fournir un meilleur soutien et des soins en ce qui concerne le suivi en temps réel de l'observance du traitement par leurs patients et éventuellement la réduction de l'attrition. Cela implique que les DAT sont une alternative appropriée au DOT pour aider les travailleurs de la santé à fournir les meilleurs soins et le meilleur soutien à leurs patients pour atteindre les objectifs de l'éradication de la TB.

TB remains the leading cause of death from infectious agents, behind only COVID-19, despite the availability of effective treatment.^1^ Directly observed therapy (DOT) has been employed globally to monitor and support adherence to TB medications. However, there is limited evidence that DOT improves treatment completion compared with self-administered therapy.^2^ Treatment completion rates remain below the 90% target in countries with the highest TB burden.^1^ In addition, DOT is associated with catastrophic costs,^3^ and rigid enforcement of DOT may conflict with the autonomy, dignity, and integrity of people with TB.^4^

Adherence to TB treatment is a major component of the WHO’s global End TB Strategy. Factors such as poor communication between patients and healthcare workers (HCWs), socioeconomic status, healthcare system factors, and treatment structures may affect medication adherence.^5^ Previous studies have indicated that non-adherence to TB medication impacts clinical and economic TB outcomes.^6^ Notably, the consequences of non-adherence include worsening of disease development and spread of drug-resistant TB.^6^

Digital adherence technologies (DATs) appear to be a promising approach for managing problems of non-adherence to medication and improving treatment outcomes. Therefore, there has been increasing interest in DATs as a more person-centred alternative to DOT. For example, DATs enable TB patients to take TB medicines at a time and place of their choice, allow HCWs to monitor adherence, and provide tailored adherence support to those who need it.^6^

DATs help manage patients with TB by monitoring and supporting their medication adherence.^7^ Several DAT platforms have been deployed, such as electronic smart pillboxes that register dosing events when the pillbox is opened,^7^ and video directly observed therapy (VDOT), whereby patients upload videos documenting medication ingestion using a smartphone or computer.^8^ These platforms have been shown to improve TB treatment success;^9,10^ however, infrastructure requirements and high maintenance costs are potential hurdles to scalability and sustainability.

99DOTS is one of the DAT models and a promising low-cost, minimal-infrastructure alternative to traditional DOT.^11^ The core component of 99DOTS is a specially designed medication blister pack or medication label that reveals a unique hidden code to be sent via SMS to a toll-free number when patients remove pills,^12^ as a proxy to determine medication intake, while VDOT patients send videos of their medication intake daily using a mobile application installed on their smart phones. For both the VDOT and 99DOTS models, an automated system records dose confirmation and aggregates patient-level adherence statistics on an adherence platform managed by Everwell Health Solutions (Bengaluru, India). Through real-time insights from the Everwell adherence platform available in desktop and mobile versions, HCWs accessed adherence data from the dashboard, using the data to identify patients struggling to adhere to treatment and a “task list” feature that provides daily summaries on HCWs to-do list for patient support. Additional support is provided to these patients through targeted SMS messaging, phone calls, and home visits.^11,12^

Although these technologies are increasingly being implemented, there is limited data on their effectiveness, acceptability, and feasibility, resulting in only a conditional recommendation for their use in TB treatment by the WHO.^13^ In addition, past evidence indicates that their effect on supporting medication adherence among patients with TB is questionable,^14^ and that engagement with patients conveys more benefits.^15^ Patients will actively engage only when they realise the benefits of engagement in healthcare.^16^

Based on the concept analysis of patient engagement, engagement in healthcare requires collaboration between HCWs and patients.^17^ In particular, HCWs should lead and foster patients to acquire awareness and confidence in engaging effectively in the care process.^18^ This highlights the crucial role that HCWs play in the success of DATs. This study explored the perception, acceptability, and challenges of DATs among HCWs.

## METHODS

### Study area and setting

The study was carried out in eight states: Benue, Kaduna, Kano, and Nasarawa in the north region and Akwa Ibom, Anambra, Imo, and Rivers in the south region. These states combined showed a treatment success rate of 90% and reflected a mix of TB burden and gaps in treatment success rate within the 14 states where KNCV Nigeria is implemented. As of 2019, a total of 5,389 DOT centres provide TB treatment services in Nigeria across primary, secondary, and tertiary levels. KNCV Nigeria, in collaboration with technical partners, implemented DATs; 99DOTS and VDOT in 98 health DOT facilities. The implementation was a pilot introduction of DAT to TB services in Nigeria.

### Study population

HCWs involved in treating patients with TB at facilities in the eight (8) implementing states during the study period who consented to participate in the DAT study. However, those who could not effectively operate smart phones were excluded.

### Study design and duration

The study was a descriptive cross-sectional study using an open-ended questionnaire hosted on Research Electronic Data Capture (REDcap) software (powered by Vanderbilt University, Nashville, TN, USA)and administered and transcribed by the National TB Programme (NTP) call centre agents to assess HCW perception, accessibility and challenges of DATs. ‘Perception’ as defined by the Britannica dictionary, is the way one thinks about or understands something, and ‘accessibility’ is the ability to be used or obtained. The study lasted for 3 months (October–December 2022).

### Sample size determination and sampling techniques

Twenty-four HCWs were purposively selected, representing 25% of the HCWs implementing DATs across the 98 health facilities. They were selected based on the level of involvement in DATs and high patient load at the facilities for equity and adequate representation. Three HCWs were studied per state.

### Data collection

Data were collected using a pretested semi-structured questionnaire adapted from the UNITAID Adherence Support Coalition to End TB (ASCENT) Study,^19^ involving open-ended questions about HCWs’ experience using DATs and their opinions on and acceptability of DATs.

### Data analysis and presentation

The quantitative data was analysed using IBM SPSS v25 (IBM, Armonk, NY, USA) and presented in tables. Categorical variables were summarised using proportion and percentages. The qualitative open-ended responses were analysed using thematic analysis in the NVivo v10 software (QSR International, Burlington, MA, USA), whereby responses were grouped into themes and subthemes and reported in prose.

### Theoretical framework

The Technology Acceptance Model (Davis, 1989),^20^ or TAM, posits that perceived usefulness (PU) and perceived ease of use (PEOU) determine whether a technology will be accepted by its potential users. In this study, the TAM was applied to identify the PU and PEOU of DATs by HCWs. Based on the model (Figure), it was assumed that if an HCW perceives the PU and PEOU of DATs, the HCW would adopt DATs to support the patients’ attitude towards medication intake, which, in turn, would improve adherence, and ultimately, lead to better treatment outcomes.

**FIGURE.**
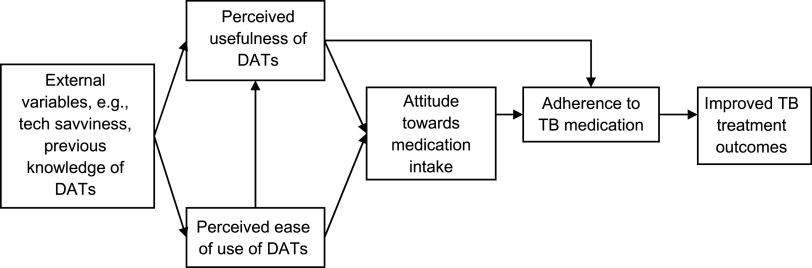
Technology Acceptance Model. DAT = digital adherence technology.

### Ethical consideration

Ethical clearance was received from the National Research and Ethics Committee (NHREC), Federal Ministry of Health, Abuja, Nigeria. Permission was obtained from the appropriate authorities. Informed consent was obtained from all participants. Voluntary participation and confidentiality were maintained.

## RESULTS

### Quantitative results

The results present understanding and use of DATs plus the opinion on the Everwell adherence platform use, which measures perception, Everwell platform use and appreciation, which measures acceptability and experience of challenges using the Everwell platform, and assesses the challenges faced by HCWs. The socio-demographics of the HCWs interviewed were as follows: 15 (62.5%) were females and 9 (37.5%) were males with a median age of 37.26 ± 8.54 years. In addition, a higher proportion (*n* = 15, 62.5%) had a tertiary degree, either a Bachelor of Science (BSc) or Higher National Diploma (HND), and the others (*n* = 9, 37.5%) had a degree from the School of Health. A higher proportion (*n* = 15, 62.5%) served as DOT focal persons and attended to 2–10 patients each day (*n* = 16, 66.6%).

Table 1 shows the understanding and use of DATs and monitoring of adherence of patients to treatment. All HCWs (*n* = 24, 100.0%) introduced DATs to all their TB patients, whereas 22 (91.7%) understood DATS and how it works; 19 (79.2%) used DATs to provide direct care, 5 (20.8%) to track summary care, and 2 (8.3%) to provide oversight. Furthermore, 17 (70.8%) patients used the Everwell platform to see if patients were taking their medications.

**TABLE 1.** Understanding and use of DAT plus monitoring of adherence of patients to treatment.

Variables	Frequency (*n =* 24) *n* (%)	
Introduce DAT to all your TB patients	24 (100.0)	
Understand DAT and how it works		
Yes	22 (91.7)	
Yes, but not much	2 (8.3)	
	Yes	No
	*n* (%)	*n* (%)
Ways you use DAT		
Provide direct care	19 (79.2)	5 (20.8)
Track summary care	5 (20.8)	19 (79.2)
Provide oversight	2 (8.3)	22 (91.7)
How you assess your patient's adherence to TB medicine		
I ask them if they take their pills when I see them	2 (8.3)	22 (91.7)
I tell them to come to the clinic so I can watch them take their pills	1 (4.2)	23 (95.8)
I talk to their family members	4 (16.7)	20 (83.3)
I talk to them on the phone	9 (37.5)	15 (62.5)
I use the Everwell platform to see if they are taking their medications	17 (70.8)	7 (29.2)

DAT = digital adherence technologies.

Table 2 shows the opinion of HCWs on the Everwell platform use. All 24 (100.0%) were positive that it was easy to explain how to use medication labels and 19 (79.2%) were positive to explain VDOT to their patients; 22 (91.7%) found it easy to identify which patients were not taking their TB medicine using DATs, and 21 (87.5) believed that Everwell adherence data helped them provide better support and counselling to their patients.

**TABLE 2.** Opinion on Everwell platform use.

Variables	Positive	Negative
	*n* (%)	*n* (%)
I received adequate training to use Everwell platform	23 (95.8)	1 (4.2)
It is easy for me to explain how to use Medication label to my patients	24 (100.0)	0 (0.0)
It is easy for me to explain how to use VOT to my patients	19 (79.2)	5 (20.8)
The 'tasklist' on the Everwell app helps me to remember to check on patients who are not taking their medicines	21 (87.5)	3 (12.5)
It is easy for me to identify which patients are not taking their TB medicine using DAT	22 (91.7)	2 (8.3)
I remember to check my patients Everwell adherence data when they come in for a refill visit	22 (91.7)	2 (8.3)
I encourage my patients to call 3340 when they have issue with DAT	23 (95.8)	1 (4.2)
Everwell adherence data helps me provide better support and counselling to my patients	23 (95.8)	1 (4.2)
My patients like using DAT	20 (83.3)	4 (16.7)
Using DAT improves the care I provide to my patients	21 (87.5)	3 (12.5)
It is easy for me or my co-workers to contact patients who have not taken their medicine	21 (87.5)	3 (12.5)
Using Everwell helps to reduce my workload	19 (79.2)	5 (20.8)
My patients who are using DAT for TB treatment visit the clinic less times than those who are not	17 (70.8)	7 (29.2)
I believe that DAT data accurately reflects if my patients took their TB medicines or not	21 (87.5)	3 (12.5)
I would recommend using DAT to my patients	24 (100.0)	0 (0.0)

VOT = video-observed therapy; DAT = digital adherence technologies.

Table 3 shows the Everwell platform use and appreciation. All 24 (100.0%) HCWs took part in the training on the use of the Everwell platform. Thirteen (54.2%) stated that training/role plays impacted their understanding of the use of the Everwell platform, followed by banners (*n* = 5, 20.8%) and (*n* = 4, 16.7%) standard operating procedures (SOPs). Most HCWs found adding patients (*n* = 17, 70.8%), followed by tracking current/past patients (*n* = 9, 37.5% each) as the most useful way of using the Everwell platform in their work.

**TABLE 3.** Everwell platform use and appreciation.

Variables	Frequency (*n =* 24) *n* (%)	
Did you take part in the training on the use of Everwell platform? Yes	24 (100.0)	
Activity/material had more impact in your understanding of use of Everwell platform		
Banners	5 (20.8)	
FAQs	2 (8.4)	
SOP	4 (16.7)	
Training/role plays	13 (54.2)	
How do you access the Everwell platform?		
Computer	1 (4.2)	
Mobile device	23 (95.8)	
Frequency of use of the Everwell platform		
Daily	21 (87.5)	
Sometimes	3 (12.5)	
How easy it is to navigate through the Everwell platform		
Easy	12 (50.0)	
Very easy	12 (50.0)	
Show patients their adherence calendar during refill visit		
No	3 (12.5)	
Yes, all	18 (75.0)	
Yes, some	3 (12.5)	
Frequency you check the 'Task list' on the Everwell platform		
Daily	15 (62.5)	
Twice a week	4 (16.7)	
Weekly	5 (20.8)	
Section(s) of the Everwell platform you found most useful to your work	Yes	No
	*n* (%)	*n* (%)
Add patient	17 (70.8)	7 (29.2)
Search patient	2 (8.3)	22 (91.7)
Current/past patient	9 (37.5)	15 (62.5)
Review VOT videos	3 (12.5)	21 (87.5)
Tasks list	―	―

VOT = video-observed therapy; DAT = digital adherence technologies.

Table 4 shows the challenges of using the Everwell platform. Thirteen (54.2%) never had any time and were not able to track their patients' adherence on the Everwell platform. Among the 11 individuals (45.8%) who encountered difficulties accessing the Everwell platform on a few occasions, the primary reasons included poor network connection or failure (*n* = 9, 37.5%), as well as (*n* = 1, 4.2% each) the lack of electricity/power, unavailability of a laptop/smartphone, and insufficient data credit.

**TABLE 4.** Experience of challenge on use of Everwell platform.

Variables	Frequency (*n =* 24) *n* (%)	
Ever been a time that you were not able to track your patients' adherence on the Everwell platform		
No	13 (54.2)	
Yes	11 (45.8)	
	Yes	No
	*n* (%)	*n* (%)
Reasons you were not able to use the Everwell platform		
No electricity/power	1 (4.2)	23 (95.8)
Poor network connection	9 (37.5)	15 (62.5)
The Everwell application or software stopped working	0 (0.0)	24 (100.0)
Did not have access to a computer or my phone	1 (4.2)	23 (95.8)
I did not have time to check the Everwell app	0 (0.0)	24 (100.0)
Lack of sufficient data credit	1 (4.2)	23 (95.8)
Contact the call centre (3340) at any point on any issue concerning the DAT project		
No	19 (79.2)	
Yes	5 (20.8)	
Reason for the call (*n =* 5)		
Technical issue	4 (80.0)	
Platform usage	1 (20.0)	
How helpful the call centre was (*n =* 5)		
Helpful	3 (60.0)	
Not helpful	1 (20.0)	
Very helpful	1 (20.0)	

VOT = video-observed therapy; DAT = digital adherence technologies.

### Qualitative results

Almost all participants agreed that the training was very useful, especially in improving knowledge and technicalities on the use of DATs. In addition, they held that DATs had facilitated their job, especially with regard to real-time tracking of their patients’ adherence to treatment and possibly reduced attrition. Equally, it impacted positively both the patients and HCWs. A few problems encountered while using the DAT models were those associated with internet availability, ownership of smart phones, and the availability of enabling networks and source of power to keep their phones charged.

## DISCUSSION

Findings from this study asserted that there was a highly positive agreement that DATs helped HCWs provide better support and counselling to their patients. They were equally positive about other anticipated uses of the platform. This implies that there is a need for DATs to support HCWs to provide the best care and support to their patients in line with the End TB goals, and that DOT was not affording them this opportunity. DOT is a standard strategy that was established to ensure adherence to treatment in the early 1990s; however, proper implementation has proved difficult to achieve worldwide. In sub-Saharan Africa, the practise of DOT is limited by a severe shortage of HCWs, coupled with the weak public health systems in which most TB programmes operate.^21^

Previous studies in Uganda have shown that only 16% of TB clinics implemented DOT properly due to a shortage of health workers.^22^ In another study, only 63% of DOT workers consistently supervised treatment.^23^ Even with modifications, such as the use of community-based strategies to enhance the implementation of DOT, including the use of volunteers, family members, or patient peers as treatment supporters, the success of such DOT models remains limited by reliance on the efforts of poorly motivated workers.^24^ Improved uptake of DATs is expected to bridge this gap.

Previous studies have shown that non-adherence can be due to forgetfulness, a false perception of wellbeing, drug side effects, stigma, long distances to health facilities, and long waiting times.^25–27^ Novel alternative approaches that address these gaps like DATs are therefore needed. A study documented that 80% of respondents have used different technologies in their respective healthcare facilities to support healthcare delivery.^28^ Other studies conducted elsewhere in comparable settings had similar findings.^29^

The reasons why HCWs were not able to use DATs on a few occasions need to be improved. In addition, training on using DATs to manage patients could have varied among these facilities, leading to different skill levels in building rapport and engaging with patients by HCWs. This needs to be standardised. Previous studies have highlighted the importance of a sufficient relationship between patients and HCWs to achieve good adherence.^30^ This critical component of patient care cannot be substituted for by the use of DATs alone. Understandably, some HCWs are more prone to motivate patients or follow-up after missed doses. Follow-up on patients could also depend on workload and location, as rural areas with frequent internet disruptions could require more follow-up by HCWs, which can be time cosuming.^31^

A meta-analysis of implementation feedback on the acceptability and feasibility of DATs reported that 99DOTS was highly acceptable to people receiving treatment for DS-TB and HCWs caring for them. However, there were some barriers that could be targets for future optimisation of these technologies and their implementation. Our study findings largely support these prior studies that demonstrated that DATs offer an acceptable, person-centred approach to TB care.^32,33^ All these studies agreed that DATs saved time and money that would have otherwise been spent travelling to clinics. Most HCWs endorsed reduced workload when using DAT.

In Addis Ababa, Ethiopia, the level of acceptability of DATs by HCWs was found to be high, and the strongest facilitator of their acceptance was perceptions of positive performance expectancy. This aligns with the WHO recognition of the potential use of technologies as discrete functionalities applied to attain health objectives.^34^ The implementation of digital health information is made possible by several factors, including trained and qualified staff, good perception of HCWs and their willingness to use the system, and adequate infrastructure.^35,36^

All HCWs studied were positive that it was easy for them to explain DATs to their patients and would recommend using DAT to their patients. In a similar study, most respondents reported that they would recommend the use of similar technology in both intensive and continuation phases of TB management.^37^ The majority in the same study viewed it as a useful alternative to DOT, especially among patients at risk of non-adherence. This acceptance is further enhanced by the COVID-19 pandemic.^37^

### Limitations of the study

A limited number of HCWs were purposively selected and studied; however, efforts were made to close this gap by ensuring that the selection of participants was based on equity and adequate representation of all HCWs representing 25% of implementing facilities. Qualitative data were collected via open-ended questions and not through in-depth interviews. This may have prevented researchers from gaining a deeper and more nuanced understanding of the barriers and facilitators to DAT implementation and acceptability.

## CONCLUSION

There is very high agreement that DATs help HCWs provide better support and care with regard to real-time tracking of their patients’ adherence to treatment and possibly reduction of attrition. They were willing to recommend DATs to their patients, and the problems encountered using the DAT models were ownership of smart phones, availability of an enabling network, and a source of power to keep their phones charged. This implies that there is a need for DATs as an alternative to DOT to help HCWs provide the best care and support to their patients so that targets towards ending TB can be achieved. However, there is a need to address the identified challenges.

## Supplementary Material


