# Supplementary material for: Perception, acceptability and challenges of digital adherence technology among TB healthcare workers
Source: Public Health Action. 2024 Jun 1;14(2):61–5. doi: 10.5588/pha.24.0008 (PMC11216288; doi:10.5588/pha.24.0008)
Supplement: Supplementary file 1 [file iutld_pha_24.0008_supplementarydata1.pdf]

http://dx.doi.org/10.5588/pha.24.0008

SUPPLEMENTARY DATA

Perception, acceptability and challenges of digital adherence technology among TB healthcare workers

Supplementary Data A

II. QUALITATIVE

Opinions about the training on using Everwell platform

Most of the participants expressed their likeness and acceptance of the training and agreed that the training was very useful especially in improving knowledge and technicalities on the use of the platform, however a small proportion erroneously expressed their opinions about the platform instead of the training as demanded by the exploratory question. They held the view that the training was able to clear all the grey areas surrounding the platform which reduced further confusion as regards the platform.

Buttressing above assertions one of these participants unequivocally stated *"…I like the training because I am now able to use the Everwell platform properly."*

And another respondent independently said "*the training on the use of the Everwell platform makes it easier …….*"

Another participant had this to say why the training was considered good: *"The training was good because it helped me to have sound knowledge on how to operate the Everwell App and also to identify patients that aren't taking their medication"*

Some of the respondents assessed the training in terms of timing, training environments and contents which they adjudged as excellent in content, inadequate in timing as one of them

declared *"...the time of the training was too short" "……because one needs a conducive environment "*

What to change about the training?

Almost all the participants accepted that there was nothing to change as regards the training as one of them stated "*There is nothing I would possibly change about the training.* "

However, a few of them noted that that the training period was short and needed to increase the days and number of staff to be trained. A participant requested *"... to increase the days used for the training, because it takes time to understand...."*

In a related manner, other respondent said: *"I will prefer that the training be extended to more than a day to help us to understand the use of the Everwell platform well.*"

And the other respondent stated: "*…. the training should be organized more than once for us to be able to understand the use of the Everwell platform better...."*

In response to what to change about the training, another trainee requested that more of the staff be trained as he said "*To train more staff members."*

How DAT has changed the work that I do since it has been implemented in my site.

Majority of the participants held that DAT had eased their job successfully especially as regards real time tracking of their patients' compliance to treatment and possibly reduce attritions. Some of the excerpts of the respondents were:

"*The implementation of DAT in my site has really improved my work speed and also helped me to follow up patients who are taking their medication and those who are not consistent in taking their medication."*

And another stated*: ".... the implementation of DAT has greatly improved our work speed and accuracy, and also helps to follow up patients who aren't adhering to their TB medicine."*

Some also argued that the introduction of DAT had increased the enrollment and service uptake in their sites as one of the participants stated:

"*…The implementation of DAT has affected my work positively in terms of patients' enrollment, and their visitation at the facility*."

In another hand, some of them believed that the introduction of DAT had actually added to their workload as one rightly stated:

"*…It actually added more workload to my work because I would have to educate and convince patient to key into DAT."*

Description of how to use Digital adherence data to manage and counsel patients.

There were diverse responses to the above exploratory leading question, however majority apart from placing calls to their clients also stated that they usually described the use of medication label codes to their patients, how to send such to the platform and the use of the charts as one of the respondents said

"...I actually use the chart to show patients how the platform works and admit to patients the importance of adhering strictly to their TB medicines."

Furthermore, some of the participants stated that managing and counselling patients using digital adherence was achieved through patient involvement and engagement as one of the participants aptly stated:

"I pick up the DAT card and read it out to the patient to let them know that their medication intake is monitored and also let them know the effect of not taking their medications."

As part of patient involvement others practically opens the platform to show thee patients the mode of operation, as one of the respondents demonstrates:

"I open the Everwell platform for patients to see outcome of sending the medication label code."

Others claimed they use it to call the patients, track, admonish and encourage the patients as one of the participants outlined,

"*……… I use it to call my patient to know why they have not been taking their medications if missed….*"

Another has this say in support *"I use to call my clients and encourage them to be consistent in taking their medication…"*

Ways DAT has been useful for the participants and description of what they like about it.

The emphasis of the usefulness of DAT and what the participants liked about it, could be considered in terms of the impact on both the patients as well as the healthcare worker. A majority of the respondents correctly argued that patient monitoring and real time tracking remained the hallmark of the DAT usefulness as some of the participants stated as follows: works.

"...DAT has really helped in the monitoring of patients' consistency in taking their medications and reduced the level in which patients default their dosages…"

Another stated *"DAT has really helped patients to adhere to their medication intake."*

Most of them stated individually: *".... It helps me monitor how my patients are taking their medications from afar……."*

The other groups who believed that the usefulness of DAT is noticed in the work itself believed that the use of DAT has facilitated the ease of working among the healthcare workers by increasing the speed, centralizing the work, as two of them declared:

"…The juicy aspect of DAT is that it helps us to monitor patients and it ease the administration of our workforce…"

"…It's now faster for the HCW to track the patients if they are taking the medication or not".

Challenges or problems encountered using the DAT models.

The problems encountered using the DAT models were those associated with the use of internet provisions including ownership of smart phones, availability of enabling network, source of energy to keep the phones charged etc, . This could be seen from two angles: from the healthcare workers end and that of the patients. Both were noted to share some of the issues in common.

From the healthcare workers end, although majority of them had a smart mobile phone but complaints of frequently poor and unstable network were noted with insufficient data as one of them stated:

"*…so far, the challenge with DAT is poor network and absence of sufficient data."*

Some of them also emphasized outdated apps as one their own major challenges as he retorted*:*
"My app was outdated so I called the person that trained us and he updated the app…."

Few others did not note any other issues aside from the tiny unreadable label prints which was said to have been resolved.

At the patients' end, a considerable proportion of these participants significantly noted that their clients reported lack of a functional smart phone, poor network availability and poor knowledge of how to send messages via a phone as one of them pointed out:

"*.. most of my clients do not have mobile phones and as such it has made it difficult for them to send the medication label code to 3340."*

On the extreme it was alluded that some of the patients dislike the platform as they detest being monitored while taking their drugs as one of the participants noted:

'*...Most patients complain that they don't want DAT, and some of them don't want the HCW to be able to track if they are taking their medication or not."*

Some of the patients were also said to complain of lack feedback as one of the participants noted *"....and secondly patients have always report at the health facility that they don't usually receive confirmation message..."*

What the participants would change if they could

A majority of the participants did not believe that there was anything to change again in DAT as one of them puts it:

"*There is nothing to change about DAT."*

Some other respondents emphasized that the DOT was much more preferred to VOT and hence should be considered more as one of them stated *"...DAT should improve more on the 99DOT than the VOT as most patients complained of not having smart phones."*

On the visibility of the label, some of the respondents would wish that the medication label be made more visible since one of them stated:

"......*Firstly, the medication label code has to be made visible enough"*

In support of this, another respondent stated:

"They find it difficult to see the number written in the scratch card."

Another area these participants believed could be changed was as it pertains to the period of sending medication label as one of the participants noted:

"*I would ensure that the medication label code is sent twice in a week and not daily."*

Some of the participants believed that medication should be taken at patients' convenience not regimented periodicity as corroborated by one of them:

"*I'll like that they use their medications at their convenient time not sticking to a particular time to take their medications."*

Provision of incentives in terms of network and data for both patients and health workers were also another area believed to be looked into as one of them highlights

"*Incentives should be given to the patients as well as the health care workers."*

A few of the participants also advocated for the establishment of support groups to reduce missed medications as he stated.

"…*to establish a group to communicate with the TB patients or any of their family member in case they miss their doses*"

Other thoughts or concerns shared about Everwell for TB patient treatment.

Almost all the participants were satisfied with the platform as majority of them individually stated ***"…****I do not have any thoughts or concerns for now."*

One other participant shared her thoughts in extending the threshold for sending the code:

"My concerns are about the missed doses because some of them take their drugs but are not able to send the code. I feel time to send the code should be extended from 24hrs to at least 48hrs."

Other comments or concerns that you want to share about using DAT.

A significant proportion of these participants had nothing to share about DAT, as stated by one of them and re-echoed by the majority:

"I do not have any other comments."

However, there was further calls for incentives and more opportunity for training and retraining of the health workers as some of them posited:

"*Health care workers should be reimbursed adequately in order to ensure effectiveness in discharging their duties."*

And another participant stated:

"*…. National TB and KNCV should put conscious effort to intensify the training they administer to health workers so as to gain them professionalism."*

The issue of insecurity and network within the facilities was also stressed as one of the respondents observed.

"…The problem of poor network, insecurity within the catchment areas where the health facilities are located should be properly address."

<u>Supplementary Data B</u>

<u>Survey Questionnaire</u>

Health Care Worker Intervention Survey
Record ID___________________________________

SECTION 1: This section should be updated after patient is enrolled on the DAT
1. Which State do you work in?
- Akwa Ibom
- Anambra
- Benue
- Imo
- Kaduna
- Kano
- Nassarawa
- Rivers

2. Akwa Ibom LGA
- Eket
- Etim Ekpo
- Etinan
- Ibeno
- Ikot Ekpene
- Itu
- Onna
- Oron
- Oruk-Anam
- Uruan
- Uyo

2 Anambra LGA
- Anambra East
- Onitsha North
- Nnewi North
- Aguata
- Anambra East
- Ayamelum
- Onitsha South
- Onitsha North

2b. Benue LGA
- Makurdi
- Gboko
- Makurdi
- Kwande
- Gboko
- Katsina Ala
- Makurdi
- Otukpo
- Konshisha
- Vandeikya
- Tarka
- Okpokwu

- Gwer East

2c. Imo LGA

- Aboh Mbaise
- Ikeduru
- Okigwe
- Orlu
- Oru East
- Oru West
- Owerri Municipal
- Owerri West

2d. Kaduna LGA

- Chikun
- Giwa
- Igabi
- Kaduna North
- Kaduna South
- Kafanchan
- Lere
- Markarfi
- Rigasa
- Sabon Gari
- Soba
- Zaria

2e. Kano LGA

- Fagge
- Garko
- KMC
- Kura
- Nasarawa
- Takai
- Tarauni
- Wudil

2f. Nassarawa LGA

- Akwanga
- Karu
- Keffi
- Lafia
- Nasarawa
- Obi
- Wamba

2g. Rivers LGA

- Abua Odual
- Ahoada
- Khana
- Obio Akpor
- Port Harcourt

3. ID of Interviewer________________________________

4. Date of Interview______________________________(This question's response should be in Gregorian calendar.)
5. Study-ID_________________________________
6. What is your gender?

- Male
- Female

7. What is your age?

- 18 - 24 years old
- 25 - 34 years old
- 35 - 44 years old
- 45 - 54 years old
- 55 - 64 years old
- 65 - 74 years old
- 75 or older

8. What is the highest educational level you have

- Have not attended school
- completed?
- Primary school not completed
- Primary school
- Secondary school
- Bachelor's degree (University) or higher
- Other (please specify)

8a. Other (please specify)________________________________
9. Which part of TB space do you work?

- Health Facility
- Other (please specify)

9a. other (please specify)_________________________________
10. Name of health facility/LGA/State office you work at?_________________________________
11. What is your role in TB care?

- Nurse
- DOT focal person
- LG Supervisor
- TB Ad hoc Staff
- Other (please specify)

(Choose as many as apply)
11a. Other (please specify)_________________________________
SECTION 2: DAT knowledge / Everwell access
12. Do you understand Digital Adherence Technologies (DAT) and how it works?

- Yes
- Yes, but not much
- No

13. Did you take part in the training on the use of Everwell platform?

- Yes
- No

13a. Which activity/material had more impact in your understanding of use of Everwell platform?

- SOP

- Training/Role Plays
- FAQs
- Banners
- Other (please specify)

13ai. Other (please specify)___________________________________
14. How do you access the Everwell platform?
- Mobile device
- Computer

15. How easy is it to navigate through the Everwell platform?
- Very Easy
- Easy
- Difficult
- Very Difficult

16. In what way do you use DAT?
- Provide direct care
- Track Summary care
- Provide oversight
- Other (please specify)
- (Check all that applies)

16a. others (Please specify)___________________________________
17. Which section(s) of the Everwell platform you found most useful to your work? (Select all that applies)
- Add Patient
- Search Patient
- Current/Past Patient
- Review VOT Videos
- Tasks List
- Other (please specify)

17a. Other (please specify)___________________________________
17b. State the reason for you answer in Q17___________________________________
18. How often do you use the Everwell platform?
- Daily
- Sometimes

19. Has there ever been a time that you were not able to track your patients' adherence on the Everwell platform?
- Yes
- No

19a. What were the reasons you were not able to use the Everwell Platform? [check all that apply]
- There was no electricity/power
- Poor network connection
- The Everwell application or software stopped working
- I did not have access to a computer or my phone
- I did not have time to check the Everwell app
- N/A
- Other (please specify)

19ai. Others (please specify)___________________________________

SECTION 3: Everwell platform/ DAT experience specific to facility direct care

20. How many TB patients do you see each day, on average?
    - 0 - 1
    - 2-5
    - 6-10
    - More than 10
21. Do you introduce DAT to all your TB patients?
    - Yes
    - No
22. How do you assess your patient's adherence to TB medicine? [check all that apply]
    - I ask them if they take their pills when I see them
    - I tell them to come to the clinic so I can watch
    - them take their pills
    - I talk to their family members
    - I talk to them on the phone
    - I use the Everwell platform to see if they are
    - taking their medications
    - Other (please specify)

22a. Other (please specify)________________________________

(Only ask the the relevant question depending onthe DAT patient is using)

23. How often do you check the 'Tasklist' on the Everwell platform?
    - Daily
    - Twice a week
    - Weekly
24. Do you show patients their adherence calendar when they come in for a refill visit
    - Yes, All
    - Yes, Some
    - No
25. Did you contact the call center (3340) at any point on any issue concerning the DAT project? Yes
    - No

25a. What was the reason for the call?
- Technical issue
- Platform usage
- Other (please specify)

25ai. Other (please specify)________________________________

25b. How helpful was the call center?
- Very helpful
- Helpful
- Not helpful
- NA

26. I received adequate training to use Everwell platform
    - Strongly Disagree
    - Somewhat Disagree
    - Neutral
    - Somewhat Agree
    - Strongly agree
27. It is easy for me to explain how to use Medication label to my patients

- Strongly Disagree
- Somewhat Disagree
- Neutral
- Somewhat Agree
- Strongly agree

28. It is easy for me to explain how to use VOT to my patients
    - Strongly Disagree
    - Somewhat Disagree
    - Neutral
    - Somewhat Agree
    - Strongly agree
29. The 'tasklist' on the Everwell app helps me to remember to check on patients who are not taking their medicines
    - Strongly Disagree
    - Somewhat Disagree
    - Neutral
    - Somewhat Agree
    - Strongly agree
30. It is easy for me to identify which patients are not taking their TB medicine using DAT
    - Strongly Disagree
    - Somewhat Disagree
    - Neutral
    - Somewhat Agree
    - Strongly agree
31. I remember to check my patients Everwell adherence data when they come in for a refill visit
    - Strongly Disagree
    - Somewhat Disagree
    - Neutral
    - Somewhat Agree
    - Strongly Agree
32. I encourage my patients to call 3340 when they have issue about DAT
    - Strongly Disagree
    - Somewhat Disagree
    - Neutral
    - Somewhat Agree
    - Strongly Agree
    - 33. Everwell adherence data helps me provide better support and counseling to my patients Strongly Disagree
    - Somewhat Disagree
    - Neutral
    - Somewhat Agree
    - Strongly Agree
34. My patients like using DAT
    - Strongly Disagree
    - Somewhat Disagree
    - Neutral
    - Somewhat Agree

- Strongly Agree

35.Using DAT improves the care I provide to my pateints

- Strongly Disagree
- patients? Somewhat Disagree
- Neutral
- Somewhat Agree
- Strongly Agree

36.It is easy for me or my co-workers to contact patients who have not taken their medicine Strongly Disagree

- Somewhat Disagree
- Neutral
- Somewhat Agree
- Strongly Agree

37. Using Everwell helps to reduce my workload Strongly Disagree
Somewhat Disagree
Neutral
Somewhat Agree
Strongly Agree

38. My patients who are using DAT for TB treatment visit the clinic less times than those who are not

- Strongly Disagree
- Somewhat Disagree
- Neutral
- Somewhat Agree
- Strongly Agree

39. I believe that DAT data accurately reflects if patients took their TB medicines or not

- Strongly Disagree
- Somewhat Disagree
- Neutral
- Somewhat Agree
- Strongly Agree

40. I would recommend using DAT to my patients

- Strongly Disagree
- Somewhat Disagree
- Neutral
- Somewhat Agree
- Strongly Agree

41. What is your opinion about your training on using Everwell platform? Please describe what you liked about it or what you didn't like about it_____________________________________

42. What would you change about the training?_____________________________________

43. Since DAT has been implemented in your site, how has it changed the work that you do?____

44. Describe how you use Digital adherence data to manage and counsel your patients?____

45. In what ways has DAT been useful for you? Please describe what do you like about it.
____

46. So far, what challenges or problems have you had using the DAT models?
____

47. If you could, what would you change about DAT?_________________________________

48. Do you have any other thoughts or concerns that you want to share about using Everwell for TB patient treatment? Please describe ____

49. Do you have any other comments or concerns that you want to share about using DAT?
